# Supplementary material for: Is parity a cause of tooth loss? Perceptions of northern Nigerian Hausa women
Source: PLoS One. 2019 Dec 5;14(12):e0226158. doi: 10.1371/journal.pone.0226158 (PMC6894835; doi:10.1371/journal.pone.0226158)
Supplement: S2 Data — (DOC) [file pone.0226158.s002.doc]

**Group 1**

**Question** 1

Is there any of you with missing /lost tooth(teeth)

**Response**

a. ….yes, I have

b. …..yes, it was extracted

c. yes, it fell on its own

d. …..yes

e….. no, I don’t

f. ……yes

**Question 2**

What do you think /know about missing/lost tooth and child bearing?

**Response**

a. ……..some women do vomit during labour and it is associated with tooth weakening and their teeth does not last long (payar baka)

b. ……..vomiting during labour is associated with tooth lost, which may be during child bearing or after child bearing

c. ……..(Payar Baka) vomiting during labour women will lose her teeth earlier (others (d and e) support the same view)

f. ………some women also have holes associated with tooth loss

**Question 3**

What is the cause of tooth loss/missing tooth in childbearing women?

**Response**

a. ……vomiting during labour (payar baka)

b. ……vomiting during labour (payar baka)

c, d and e - same as above

f. …. cancer, big or small makes the teeth to be shaking and fall after some time

**Question 4**

What are the effect of missing /lost tooth on life of a woman?

**Response**

a. …..No effect

b. ...anytime I try to chew meat or maize, it is discomforting when the food is on the gum at the back where there are no teeth. I don’t enjoy it.

c. …. not be beautiful and can’t laugh well

d. … difficulty in eating/ chewing (I am unable to chew meat or maize, I miss out on them)

e. …..no effect

f…. no effect (cause women is already old)

**Question 5**

Between men and women who lose more teeth?

**Response**

………women (unanimously/All agreed)

Question - Why?

Reason: …….during childbirth due to vomiting during labour

**Question 6**

Is there any relationship between childbearing and tooth loss?

**Response**

a……..Yes. I think because I know a woman with five children and she lost some teeth.

b, c, d and e - ….Yes, due to vomiting during labour, which leads to weakening of the tooth (payar baka).

f …….No, because some women did not experience vomiting and not associated with tooth loss

Question -Is there any association between food consumption during pregnancy and tooth loss?

**Response**

a. …..No, it is not associated with any food consumption during pregnancy

b.…..it is because the woman is unable to take care of her teeth or due to consumption of some food

d. ……..No relationship with the food women eat

**Question 7a**

How many of you lost/missed teeth during child bearing and how many teeth?

**Response**

a. …..no

b. …………vomiting during labour in women, which will make the teeth weak and will not last. Yes, I am having vomiting during labour and only one tooth lost after menopause

c. ….Yes, I do vomit during labour and I lost my tooth (two teeth) –payar baka

Question - Is there anyone who lost teeth before the first child was born ?

**Response**

……No (all)

**Question 7b**

Is there any relationship between the number of children and the number of teeth lost (the number of children women have and the number of teeth lost is there any relationship)?

**Response**

……. No (all)

Hypothetical case

Question - I have two friends and one of them lost two teeth and has 4 children and the other one has no children and has not lost any teeth. What do you think?

**Response**

……Maybe the woman that lost teeth has cancer or she vomits during pregnancy

**Question 8**

Is there any saying/proverb/adage on child bearing and tooth lost?

**Response**

…….No saying except the belief that if a woman is vomiting during labour she is going to loss her teeth (unanimously)

**Group 2**

**Question 1**

Is there any of you with missing/lost teeth(tooth)?

**Response**

a. …..Yes, I have extracted one of my teeth

b. ….yes, it removed on its own

c. …..yes, it broke in pieces

d. …..yes, I lost 1(one) due to trauma while 4 (four) teeth lost on their own

e. …..yes, it removed on its own

f. …….yes, I had one extracted

**Question 2**

What do you think /know about missing/lost tooth and childbearing?

**Response**

a. ……it started with pain and was later extracted

b. …. my tooth was removed because of pain. There was a hole in it

c. ……tooth was shaking and later removed on its own

d. ……I had it extracted because it was broken

e. ….. it fell by itself

**Question 3**

What is the cause of tooth loss/missing tooth in childbearing women?

**Response**

a. ….hole made by tooth worms

b. …tooth worms destroy the teeth

c. ……tooth worms eat the tooth and make a hole in it, this cause pain

d. ….drinking cold water

e. ….Headache

f. ….drinking cold water and cold weather will cause pain in the tooth

**Question 4**

What are the effects of missing /lost tooth on life on the life of a woman?

**Response**

a.…..unable to chew and to replace with artificial teeth you have to pay money (financial burden)

b. …… I can’t laugh well because the gap will show

c. …..because it is front teeth I don’t have difficulty in chewing but I don’t feel pretty like I was when the teeth were there.

d. …..I don’t like to laugh or talk in public so that people will not laugh at me

e. ……unable to smile because its front teeth

**Question 5**

Between men and women who lost more teeth?

**Response**

a. …women

b. ……..women become older than men and it is because of childbearing. Because of this women lose more teeth

c. ……..women

d. …..women

e. …..not sure

f. …….not sure

**Question 6**

Is there any relationship between childbearing and tooth loss?

**Response**

……..No relationship (unanimously)

**Question 7a**

How many of you lost/missed teeth during child bearing and how many teeth?

**Response**

b…….. I vomited when I was going to have my last child and my tooth fell off (payar baka)

…….no (others)

**Question 7b**

Is there any relationship between the number of children and the number of teeth lost (the number of children women have and the number of teeth lost is there any relationship)?

**Response**

f……..I lost some teeth before I marry so it cannot be pregnancy that cause it since I was not pregnant before I marry

No (Unanimously)

**Question 8**

Is there any saying/proverb/adage on child bearing and tooth lost?

**Response**

…………No (unanimously)

**Group 3**

**Question** 1

Is there any of you with missing /lost tooth (teeth)

**Response**

c. …….yes, I had hole and later I had guava piece impacted into the hole

d. ……yes, I lost a tooth

e. …….yes, I have missing teeth

…..No (others)

**Question 2**

What do you think /know about missing/lost tooth?

**Response**

a. No response

b. …..as you grow old the teeth are no more strong, they are weak and will fall out one by one

**Question 3**

What is the cause of tooth loss/missing tooth?

**Response**

a. ……..dirty mouth have worms that damage the teeth

b. ….tooth worms

c. ……poor personal hygiene make the mouth to smell after sometime the teeth will be shaking and fall out

d. …….poor oral hygiene, old age (aging), trauma will make the teeth weak and loose

e. ….old age/aging

f. ……dirty mouth

**Question 4**

What are the effects of missing /lost tooth on the life of a woman?

**Response**

a. …….you don’t look beautiful again

b. …… it affects the beauty when you lose your front teeth you don’t look beautiful again

c. ….affects beauty and you don’t feel good

d. …….. it affects her beauty

e. ….unable to smile

**Question 5**

Between men and women who lost more teeth?

**Response**

……..women (Unanimously)

Reasons

a. ….vomiting during labour in female, which will make the teeth weak and will not last (payar baka)

b. …….women become old earlier than men and it is because of childbearing

**Question 6**

Is there any relationship between childbearing and tooth loss?

**Response**

a. ……yes, there is a relationship

Reasons

a. …….because of vomiting during labour (payar baka)

b. …….no relationship

c. …… it is due to cancer

d. ……pregnancy will not cause tooth loss, tooth loss is due to tooth ache and fracture

e. …….no relationship between childbearing and tooth loss

**Question 7a**

How many of you lost/missed teeth during childbearing and how many teeth?

**Response**

d. ……….I lost two teeth

e…. I lost a tooth because I vomited when I was in labour (payar baka)

Question-Any relationship to pregnancy or during childbirth

d. ……no, it is not during pregnancy, it is due to guava impaction

f. ……..I have 12 children without losing any tooth

**Question 7b**

Is there any relationship between the number of children and the number of teeth lost (the number of children women have and the number of teeth lost is there any relationship)?

**Response**

f. ……It’s not due to pregnancy. It’s due to guava seed impaction between the teeth but that happened during pregnancy, I have 12 children without losing any tooth.

…..No (unanimously)

Hypothetical aid; I have two friends

1. Married with 4 children and she lost 2 teeth

2. Married with a child and lost no teeth

No relationship between number of children and tooth lost –

d. ….. my friend who left the village now has missing teeth may be because she works more and she is not able to take care of her body. She is going to become very old earlier than people in the city.

f. …… maybe she chews gums which contains sugars and can cause hold and tooth loss.

**Question 8**

Is there any saying/proverb/adage on child bearing and tooth lost?

**Response**

….No (All agreed there is none)

**Group 4**

**Question** 1

Is there any of you with missing /lost tooth(teeth)

**Response**

…No (all)

**Question 2**

What do you think /know about missing/lost tooth?

**Response**

No response

No idea

**Question 3**

What is the cause of tooth loss/missing tooth?

**Response**

a. ……cancer is a big problem that eats the teeth and mouth

b. …….people fight can hit each other on the teeth with a blow, when you fall down you teeth can break or come out

c. …..old age / aging

d. ……Okada (motorcycle) or car accidents can make teeth fall out if you hit you mouth on the Okada or ground

e. … lack of brushing and the teeth will fall out

f. ……consumption of sugar coating food makes hole in teeth

**Question 4**

What are the effects of missing /lost tooth on the life of a woman?

**Response**

a. …….difficulty in feeding

b. …it affects beauty and difficulty in chewing

c. ……you don’t look beauty

d. ……affects beauty

e. …..affects beauty

f. ….. affects beauty and feeding problem

**Question 5**

Between men and women who lose more teeth?

**Response**

a. ………women-because of poor oral hygiene and kola nut chewing

b. …….men -because they get in fight and road traffic accident a lot

c. ……men do not clean their teeth very well

d. ……men

e. …..men

f. …..women

**Question 6**

Is there any relationship between childbearing and tooth loss?

**Response**

a. ….No idea

b. …..no idea

c, …we heard that when women are having vomiting during labour their teeth are not going to last. (payar baka)

d. ……..vomiting during labour can cause tooth loss

e. …….vomiting during labour will cause tooth loss (payar baka)

f. …….vomiting during labour can cause tooth loss

**Question 7a**

How many of you lost/missed teeth during child bearing and how many teeth ?

**Response**

……….No (all)

**Question 7b**

Is there any relationship between the number of children and the number of teeth lost(the number of children women have and the number of teeth lost is there any relationship)?

**Response**

……..No (unanimously)

……..……some women also have holes in teeth associated with tooth loss but not really associated with childbearing. Because even a very young girl can have holes in teeth associated tooth lost.

**Question 8**

Is there any saying/proverb/adage on child bearing and tooth lost?

**Response**

…..we heard that women are vomiting during labour their teeth are not going to last

……when women are vomiting during labour their teeth are going to be weak

**Group 5**

**Question** 1

Is there any of you with missing /lost tooth (teeth)

**Response**

e. …….yes, I lost a tooth (only one participant)

Reason ….Yes, my tooth came out on it own. It was shaking before it fell off

**Question 2**

What do you think /know about missing/lost tooth?

**Response**

a. …….the tooth is very painful before it removes

b. …….missing tooth very painful and it gives headache

c. ……very painful and I can not chew properly

d. ….. painful

e. ……No idea

**Question 3**

What is the cause of tooth loss/missing?

**Response**

a. ………cancer is a swelling that make the teeth to fall out

b. ………some women are unable to take care of their teeth because they do not care about their mouth

c. …….. may be cancer, or you fall down and hit your mouth on the ground and dirty mouth

d…… I hear traditional medicine people say that worms makes hole in teeth and later gives pain and tooth will be removed

e. ……dirty mouth

f. ……punch or slap on the face from fights between two people can make teeth fall off

**Question 4**

What are the effects of missing /lost tooth on life of the woman?

**Response**

a. …….beauty is lost and chewing difficulties

b. ……the person will not look beautiful again

c. ……unable to chew food/meat

d. …..difficulty in speaking and unable to chew

e. …..I cannot laugh very well and have difficulty in chewing

**Question 5**

Between men and women who lost more teeth?

**Response**

Women (unanimously)

**Reason**

………..girls like to eat sweets and chew gum , these make their teeth go bad . They cause holes and pain and the teeth are removed later.

**Question 6**

Is there any relationship between childbearing and tooth loss?

**Response**

a. …..yes, due to vomiting during labour (payar baka)

b……. I heard an old woman saying that vomiting during labour will lead to weakening of teeth

Question -Do you believe in that/ what’s your perception on that?

…..Yes, we believe

……I believe (unanimously)

**Question 7a**

How many of you lost/missed teeth during child bearing and how many teeth?

**Response**

a.……..Yes (One participant - one tooth only)

b... I vomited during labour but did not lose a tooth but I am afraid that I will lose one soon or later

The remaining participants-No

**Question 7b**

Is there any relationship between the number of children and the number of teeth lost (the number of children women have and the number of teeth lost is there any relationship)?

**Response**

……No (unanimously)

**Question 8**

Is there any saying/proverb/adage on child bearing and tooth lost?

**Response**

a, ……Yes, we heard old women saying that if a woman experience vomiting during child labour she will lose her tooth /teeth during childbirth or after menopause (payar baka)

b. ……yes, we heard old women saying that if a woman experience vomiting during child labour she will lose her tooth /teeth during childbirth or after menopause

c. …..yes, we heard old women saying that if a woman experience vomiting during child labour she will lose her tooth /teeth during childbirth or after menopause

d. ………No

e. …..yes, we heard old women saying that if a woman experience vomiting during child labour she will lose her tooth /teeth during childbirth or after menopause

f. …..no

**Group 6**

**Question** 1

Is there any of you with missing /lost tooth(teeth)?

**Response**

d…….. yes, I lost some teeth

**Question 2**

What do you think /know about missing/lost tooth?

**Response**

a. ………..yes I heard people saying that if a woman is vomiting during labour she will loss her tooth early in life.

b. …..don’t know about it

c,…. No idea

d. ….no idea

e. …..no idea

f. ………don’t now about it

**Question 3**

What is the cause of tooth loss?

**Response**

a. …….. meat impaction between teeth

b. *…..…….*using teeth to open drink/ Coca-Cola crown tops or biting something hard. The tooth can break or fall out

c. …..hole and tooth worms

d. …….chewing bones will break your teeth

e. …….tooth worms make hole in teeth

f. .... drinking cold water and cold weather

**Question 4**

What are the effects of missing /lost tooth on the life of a woman?

**Response**

a. ….you cannot eat some food

b. ……. very painful associated with missing of joy because you cannot talk or laugh like you want to in public

c. ……difficulty in chewing and not beautiful like before

d. …...difficulty in chewing meat and maize

e. …affects beauty

f. ……affects beauty

**Question 5**

Between men and women who lose more teeth?

Answer

….women (unanimously)

**Question 6**

Is there any relationship between childbearing and tooth loss?

a. …..Yes, due to vomiting during pregnancy and labour

b. …..some women experience vomiting during pregnancy and labour and are associated with tooth ache and tooth loss

**Question 7a**

How many of you lost/missed teeth during child bearing and how many teeth?

a. …. I know that pregnancy will cause lose of hair, nail, tooth. It also causes pain in tooth. When I was pregnant I lost my hair but not my tooth or nail

No (others)

**Question 7b**

Is there any relationship between the number of children and the number of teeth lost (the number of children women have and the number of teeth lost is there any relationship)?

**Response**

a. ……..no

b. …….yes, because I think I know a woman with 5 children and lost some teeth

c…….. I don’t know

d. . …….I lost some teeth before I marry or even have children. It cannot be pregnancy maybe the sweets and chewing gum I took when I was young

e. …….I don’t think so

f. …………I don’t know

**Question 8**

Is there any saying/proverb/adage on child bearing and tooth lost?

**Response**

a. ……..no I don’t know

b. …… the number of pregnancy that a woman has is the number of teeth she will lose.

c. ….no I don’t think so

d………. yes I heard about it that pregnancy will cause loss of hair, nail and tooth

e. ………I don’t think so/ I don’t know

f. ……..I don’t know
